# Discovery and Development of the Enantioselective Minisci Reaction

**DOI:** 10.1021/acs.accounts.3c00247

**Published:** 2023-07-05

**Authors:** P. David Bacoş, Antti S. K. Lahdenperä, Robert J. Phipps

**Affiliations:** Yusuf Hamied Department of Chemistry, University of Cambridge, Lensfield Road, Cambridge CB2 1EW, United Kingdom.

## Abstract

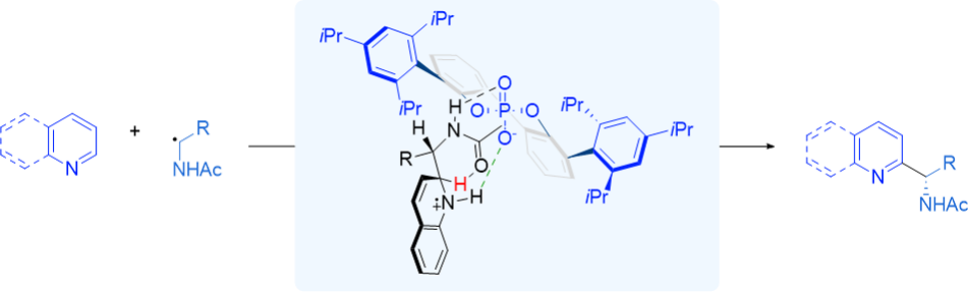

The class of reactions now known as Minisci reactions is broadly defined as the addition of nucleophilic carbon-based radicals to basic heteroarenes with subsequent rearomatization to form a new carbon–carbon bond. Since the pioneering work of Minisci in the 1960s and 1970s, these reactions are now widely used in medicinal chemistry due to the ubiquity of basic heterocycles in druglike molecules. One of the long-standing challenges of Minisci chemistry has been that of regioselectivity due to the mixtures of positional isomers commonly obtained on many substrates if there is a choice between similarly activated sites. At the outset of the work described herein, we hypothesized that it may be possible to tackle this using a catalytic strategy whereby a bifunctional Brønsted acid catalyst simultaneously activates the heteroarene and engages attractive non-covalent interactions with the incoming nucleophile, resulting in a proximal attack. Using chiral BINOL-derived phosphoric acids, we not only were able to achieve this goal of regiocontrol but also discovered that we could control the absolute stereochemistry at the new stereocenter formed when prochiral α-amino radicals were employed. At the time, this discovery was unprecedented in the context of Minisci reactions.

This Account details the discovery of this protocol and the further development, expansion, and investigations into the mechanism that we have carried out since then, several in collaboration with other research groups. Collaborative efforts have involved an expansion of the scope to diazines guided by multivariate statistical analysis through the development of a predictive model (collaboration with Sigman). Also, a mechanistic study involving detailed DFT analysis (collaboration with Goodman and Ermanis) unveiled the selectivity-determining step as being the deprotonation of a key cationic radical intermediate by the associated chiral phosphate anion. We have additionally carried out a number of synthetic developments of the protocol such as removing the need to prefunctionalize the radical nucleophile; hydrogen-atom transfer can be used to enable a formal coupling of two C–H bonds to form a C–C bond while retaining high enantio- and regioselectivity. Most recently, we have been able to expand the protocol so that α-hydroxy radicals can be used: until this point, all examples had concerned α-amino radicals. Again, HAT was used to generate the α-hydroxy radicals, and DFT studies carried out in collaboration (Ermanis) provided mechanistic insights.

Since our original report, there have appeared a number of exciting developments from other research groups whereby the protocol has been applied to new substrates or using different precursors to generate the requisite α-amino radical. There have also been several examples in which alternative photocatalyst systems have been used to reduce the redox-active esters in the original enantioselective Minisci protocol. While primarily an Account, these contributions from other research groups will be covered briefly for context toward the end of the article.

## Key References

- ProctorR. S. J.; DavisH. J.; PhippsR. J.Catalytic enantioselective Minisci-type addition to heteroarenes. Science2018, 360, 419–42210.1126/science.aar6376.29622723(1)*The first report of an enantioselective Minisci reaction.*
- ErmanisK.; ColganA. C.; ProctorR. S. J.; HadrysB. W.; PhippsR. J.; GoodmanJ. M.A Computational and Experimental Investigation of the Origin of Selectivity in the Chiral Phosphoric Acid Catalyzed Enantioselective Minisci Reaction. J. Am. Chem. Soc.2020, 142, 21091–2110110.1021/jacs.0c09668.33252228PMC7747223(2)*We carry out mechanistic experiments and collaborate with computational colleagues Prof. Jonathan Goodman and Dr. Kristaps Ermanis to gain insight into the origin of selectivity in the reaction.*
- ProctorR. S. J.; ChuentragoolP.; ColganA. C.; PhippsR. J.Hydrogen Atom Transfer-Driven Enantioselective Minisci Reaction of Amides. J. Am. Chem. Soc.2021, 143, 4928–493410.1021/jacs.1c01556.33780237PMC8033566(3)*In this work, we demonstrate that it is possible to move away from prefunctionalized radical precursors such as redox-active esters and generate the required α-amino radical through simple HAT. Furthermore, no added photocatalyst is required.*
- ColganA. C.; ProctorR. S. J.; GibsonD. C.; ChuentragoolP.; LahdenperäA. S. K.; ErmanisK.; PhippsR. J.Hydrogen Atom Transfer Driven Enantioselective Minisci Reaction of Alcohols. Angew. Chem., Int. Ed.2022, 61, e20220026610.1002/anie.202200266.PMC932172135420220(4)*We demonstrate that it is possible for α-hydroxy radicals to be used as nucleophiles, with the hydroxy group hydrogen bonding with the chiral phosphate during enantiodetermining deprotonation.*

## Introduction

**Figure 1:**
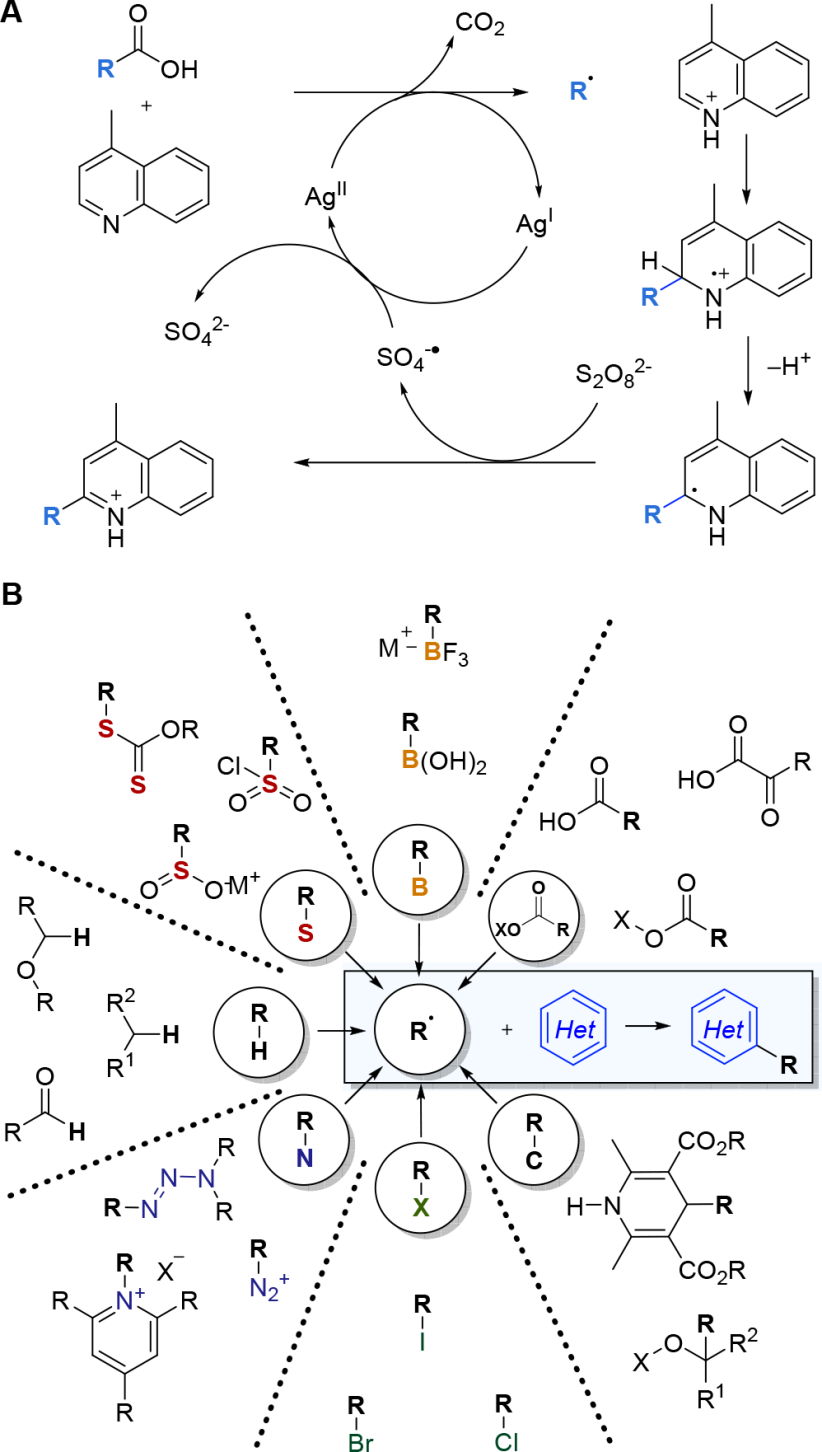
A: Original silver-catalyzed decarboxylative protocol was developed by Minisci. B: Examples of radical precursors that are now used in Minisci reactions.

The Minisci reaction, as it is often referred to, originated from Francesco Minisci’s pioneering work on the addition of nucleophilic, carbon-centered radicals to electron-deficient heterocycles, a research effort that commenced in the 1960s and continued with remarkable productivity throughout subsequent decades.^5^ Minisci made important contributions to numerous areas of radical chemistry, but it is with this basic reactivity pattern that his name is most associated. Since that time, Minisci-type reactions have become widespread as a versatile tool for elaborating basic heteroarenes, in no small part driven by the fundamental importance of these motifs in medicinal chemistry.^6^ The emergence of photoredox catalysis in the past decade as a powerful and extremely convenient tool to permit the generation and relay of reactive radical intermediates has brought about renewed interest in Minisci chemistry.^7^ The original Minisci protocol for radical generation which involved decarboxylation promoted by stoichiometric silver salts (Figure 1A), added to gradually over the years, has now been joined by countless radical generation methods using a variety of precursors, and the reaction type has become something of a testing ground for new radical generation protocols (Figure 1B).

**Figure 2:**
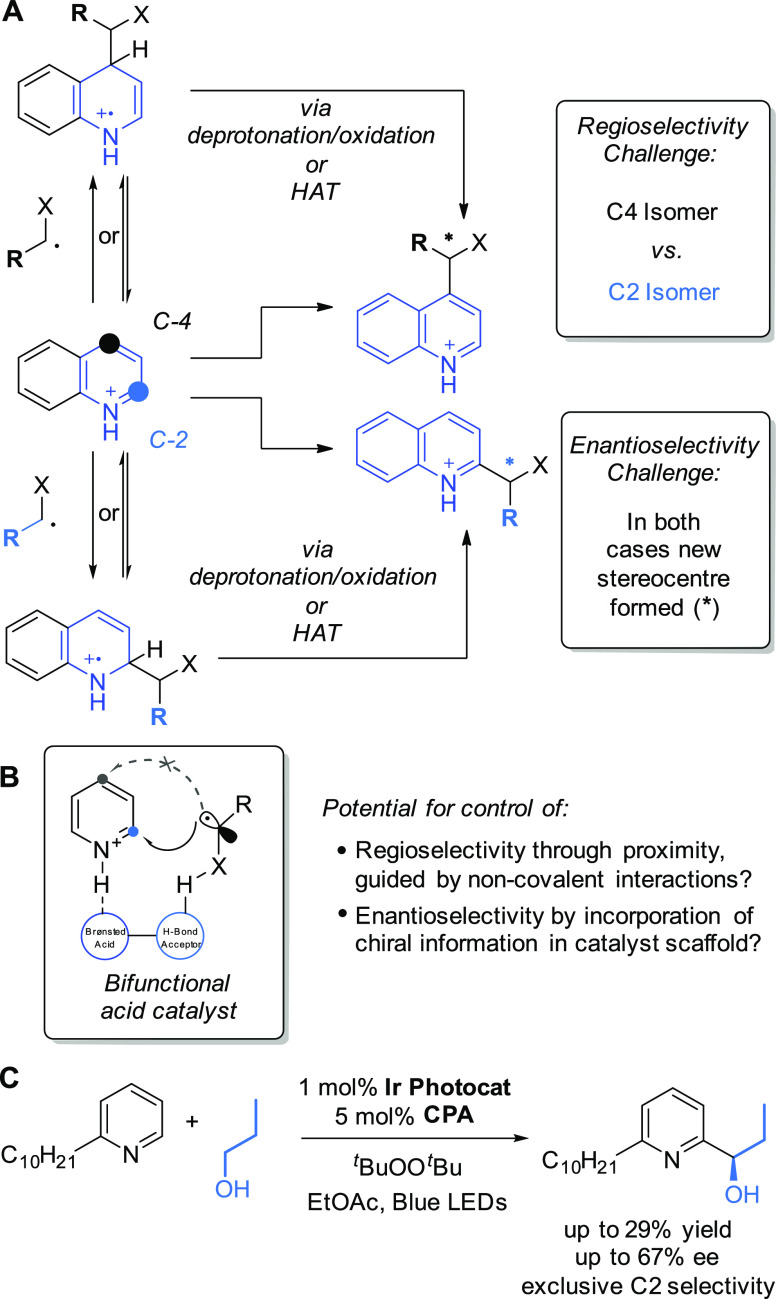
A: Unaddressed selectivity challenges in Minisci-type reactions. B: Hypothesis at the outset of this work. C: Summary of our initial encouraging results obtained using α-hydroxy radicals produced through HAT.

Our initial interest in Minisci-type reactions was not related to radical generation but rather the selectivity challenges associated with the reaction. The most prominent and obvious was that of regioselectivity, specifically the propensity to form regioisomers when several electronically similar positions are available on a given heteroarene. This arises due to several LUMO coefficients of a protonated heteroarene often being so similar that there may be little discrimination between them.^6d^ An archetypal example of this challenge is that quinoline will typically give rise to a mixture of monofunctionalized C2 and C4 isomers as well as a difunctionalized product, a problem that can be circumvented only by blocking one of the two positions (Figure 2A). This challenge extends to any heteroarene which has electronically similar positions and thus represents a major drawback to the wider application of Minisci-type reactions. Conscious of this issue, Minisci and co-workers carried out important studies into the effects of factors such as solvent polarity and acidity,^8^ and O’Hara, Blackmond, and Baran later reported careful studies empirically rationalizing prediction of regioselectivity.^9^ A second, perhaps less obvious, selectivity challenge associated with Minisci-type reactions is that of absolute stereochemistry, if a prochiral radical nucleophile is utilized. In such a situation, a new stereocenter will certainly be formed in the product, but all approaches at the outset of the work described herein formed only a racemic mixture. This type of mixture is of less immediate inconvenience to the organic chemist than a regioisomeric mixture, so this selectivity challenge has probably been less widely considered. But the potential of combining a reaction that directly forges a C–C bond onto a basic heteroarene with a protocol that can also control an adjacent stereocenter would be of formidable utility, particularly in the synthesis of pharmaceutically relevant molecules.

## Discovery of the Enantioselective Minisci Reaction

At the outset of the work described herein, we envisaged a strategy, based on catalysis, that could feasibly enable the control of regioselectivity in Minisci reactions but would also have the potential to influence enantioselectivity if prochiral radicals were used. We hypothesized that a bifunctional catalyst may use its acidic functionality to protonate, and thus activate, the heteroarene while the second functionality would engage in an attractive non-covalent interaction, such as a hydrogen bond, with a suitably functionalized nucleophilic radical (Figure 2B). Such an arrangement may be expected to position the radical in close proximity to the C2 position of the activated heteroarene and thus control the regioselectivity in the addition. If the catalyst were chiral and enantiopure, then the possibility may also exist for control of enantioselectivity in the newly formed stereocenter if a prochiral radical is used. This proposal, as outlined, implies that radical addition would be the selectivity-determining step. But given that the conjugate base of the hypothetical catalyst would likely remain associated with the resulting radical cation following radical addition, it may have the ability to influence later steps of the mechanism which may be selectivity-determining, such as deprotonation (see Figure 2A).

**Scheme 1:**
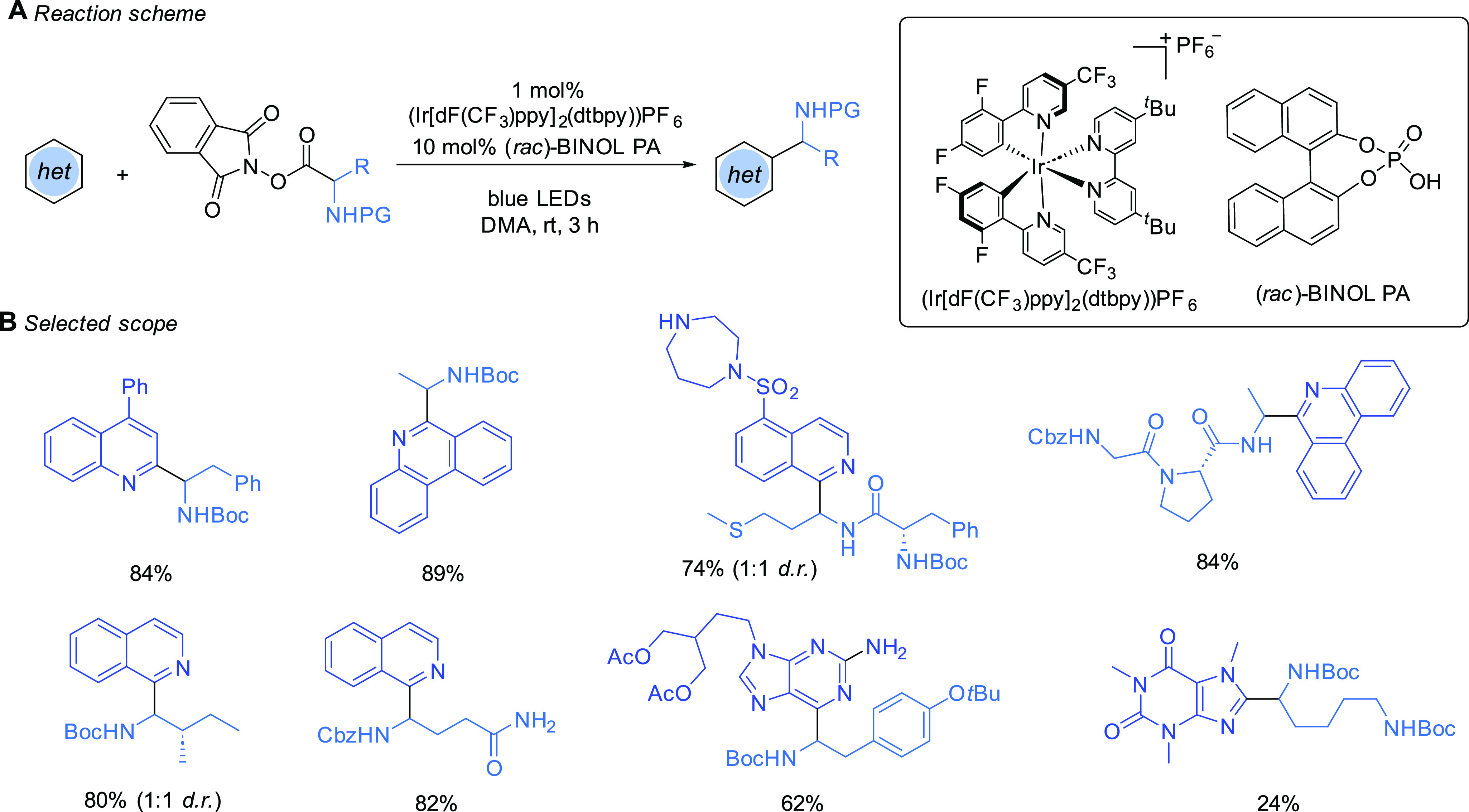
Use of Amino Acid-Derived Redox-Active Esters as Radical Precursors in Minisci Reactions, as Reported by Shang, Fu, and Co-workers

We quickly realized that organic phosphoric acids should fulfill our outlined requirements of a bifunctional catalyst very nicely. Rather than necessitating a bespoke design with two separate functional groups, the phosphoric acid group encompasses both—Brønsted acidity (POH) combined with an excellent hydrogen bond acceptor in the phosphoryl oxygen (P=O).^10^ Over the past two decades, organic phosphoric acids have risen to prominence as privileged chiral catalysts for diverse applications in asymmetric synthesis, initiated by the seminal reports of Akiyama and Terada using BINOL-derived chiral phosphoric acids (CPAs).^11^ A BINOL-derived CPA had at that point been successfully used in combination with photoredox catalysis in influential work from Knowles and co-workers whereby enantioselectivity is controlled in a radical cyclization onto a hydrazone.^12^ This was enabled by the chiral phosphate engaging in hydrogen bonding interactions with the intermediate α-hydroxy radical.^13^ Using a non-CPA catalyst, an important study from Ooi and co-workers demonstrated the power of a combination of hydrogen bonding and ion pairing to control enantioselectivity in a radical–radical coupling.^14^ We began by investigating whether catalytic amounts of TRIP, the archetypical example of a CPA, could promote the addition to pyridine of α-hydroxy radicals obtained through hydrogen atom transfer (HAT) from simple alcohols. From those early studies, we were very much encouraged by observations of high regioselectivity as well as promising levels of enantioselectivity, although reactions were hampered by low product yields seemingly resulting from byproducts arising from the radical generation conditions employed (Figure 2C). Crucially, catalytic amounts of TRIP were effective and both selectivity aspects could be positively impacted, demonstrating conclusively that the reaction mechanism was amenable to catalyst control in this way. Around this time, Shang, Fu, and co-workers demonstrated in two important publications that redox-active esters (RAEs) could be used in combination with photoredox catalysis to generate alkyl radicals for use in Minisci reactions (Scheme 1).^15^ The first of these specifically dealt with the generation of amino acid-derived α-amino radicals, typically *N*-Boc-protected and in DMA as solvent, and exhibited a broad scope with respect to both reaction components.^15a^

**Scheme 2:**
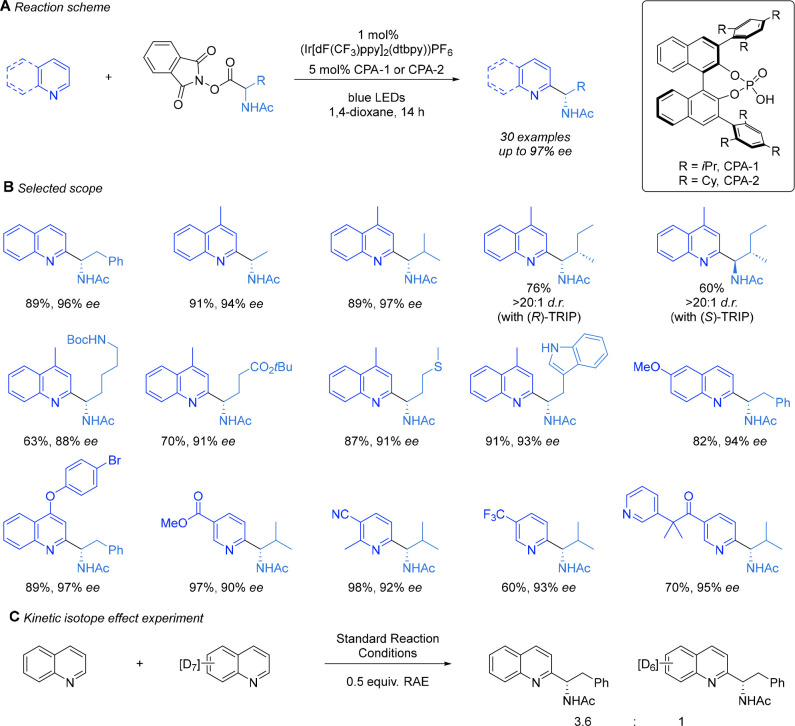
Summary of the Scope of the Enantioselective Minisci Reaction Using Chiral Phosphoric Acids and RAEs as Radical Precursors

This caught our attention, as the α-amino radicals, in which the nitrogen atom was typically *N*-Boc-protected, possessed hydrogen bond donor functionality analogous to that of the α-hydroxy radicals we had been investigating. We decided to evaluate the α-amino radicals using the Shang/Fu RAE protocol but using TRIP (referred to as CPA-1 in the schemes) as a Brønsted acid catalyst, analogous to our encouraging initial investigations with alcohols. (We subsequently returned to the challenge of using alcohols as radical precursors, detailed later in this Account.) After brief optimization, we found that use of α-amino radicals in which the nitrogen atom was *N*-acetyl-protected resulted in exclusive C2 regioselectivity in nonpolar solvents such as dioxane (Scheme 2A). Furthermore, excellent enantioselectivity in the newly formed stereocenter was observed.^1^ The *N*-acetyl group was found to be of great importance; the *N*-Boc analogue, for example, under the same conditions gave both a low yield and low ee. The scope of the transformation in terms of both the heteroarene and the amino acid-derived RAE (precursor to the *N*-acyl, α-amino radical) was explored (Scheme 2B). The RAEs were readily derived from commercially available *N*-acetyl amino acids, and we observed that, for the most part, the amino acids were racemized during the synthesis of the RAE; only in a few hindered examples was this not the case. The reaction conditions were very tolerant of functionality, and the scope of this component was broad, tolerating side chains including a thioether, N–H indole, Boc-protected amines, and an ester. This tolerance to the photoredox catalysis conditions had been expected, based on the precedent of Cheng, Shang, and Fu, but we were very happy to see the excellent enantioselectivity values obtained for such a range; small substituents such as methyl were well tolerated, alongside far bulkier ones such as isopropyl. Full diastereocontrol could be obtained using either (*R*)-TRIP or (*S*)-TRIP when a RAE derived from l-isoleucine was employed. For the heteroarene scope, we surveyed a range of substituted and elaborated quinolines and continued to obtain excellent regioselectivity (>20:1 for C2) and enantioselectivities (Scheme 2B). We also found that the protocol was amenable to pyridines, with the caveat that the pyridine must possess an electron-withdrawing substituent somewhere other than the 6-position. For these, we found that the bulkier TCYP catalyst (referred to as CPA-2 in the schemes) gave slightly higher ee values than TRIP. Several applications of the reaction to more complex substrates were carried out, and Metyrapone, an inhibitor of cortisol biosynthesis, is a particularly appealing example because it contains two 3-substitued pyridines and potentially six different sites at which reaction could occur. Using our protocol, a single isomer could be obtained in 95% ee along with a good yield (70%) with no traces of other isomers, a powerful demonstration of the extremely high level of control over multiple selectivity aspects imparted by the CPA catalyst. We carried out some initial mechanistic probe experiments, including an intermolecular competition KIE experiment between quinoline and quinoline-*d*_7_ (Scheme 2C). This resulted in a primary KIE of 3.6, implying that the C–H cleavage occurs in the product-determining step of the reaction, which, in this case, would most likely be deprotonation of the radical cation following radical addition (Figure 2A). This implies that the initial radical addition is reversible, an observation which agreed with those of Minisci, who, using similar experiments, identified that the radical addition is commonly reversible for stabilized radical nucleophiles, in that specific case α-oxy radicals derived from ethers.^8^ We also observed a positive non-linear effect under the optimized conditions, to be discussed in more detail later.

In subsequent related non-enantioselective work, we surveyed a range of achiral Brønsted acids in various solvents with the aim of identifying conditions to allow regiocontrol between the C2 and C4 products for the same combination of reactants. This proved partially successful, and synthetically useful levels of regioselectivity for either C2 or C4 could be obtained, although the yields in many cases were modest and the C2 selectivity was not at the extremely high levels seen when TRIP was used as the catalyst.^16^

## Development of the Enantioselective Minisci Reaction

**Scheme 3:**
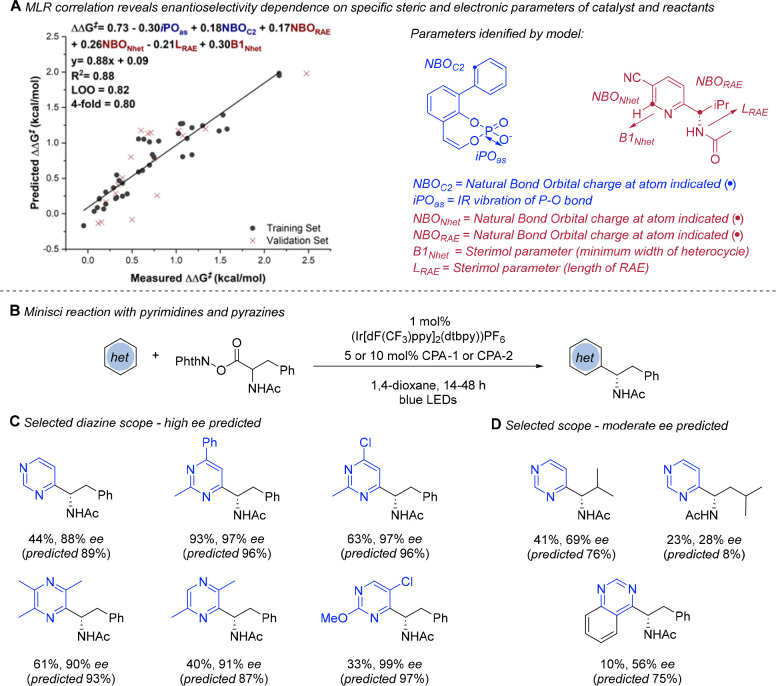
Application of the Multivariate Statistical Analysis Approach to Developing a Predictive Model for the Enantioselective Minisci Reaction and Extension of Scope to Diazines Panel A is reproduced with permission from ref (19), copyright 2019 American Chemical Society.

In early 2018, we began collaborating with the group of Prof. Matthew Sigman (University of Utah). The Sigman group had already established a unique program whereby statistical analysis is applied to focused data sets from catalytic enantioselective reactions, with the aim of developing predictive models to accelerate reaction and catalyst development.^17^ Some of the foundational work of that program had involved the analysis of reactions catalyzed by CPAs, in collaboration with the Toste group.^18^ The Sigman group was able to utilize its existing expertise and parametrization efforts for these catalysts and apply it to the enantioselective Minisci reaction. This involved working closely together to assemble a designed data set in which both the substrate and catalyst were systematically modified with the intention of identifying effective correlation with steric and electronic parameters that would allow an accurate prediction of the suitability of unknown substrates.^19^ Furthermore, from the scope we had several instances of poorly performing substrates, in terms of ee, which were difficult to rationalize and which we anticipated would provide interesting fuel for a data-driven approach. An iterative multivariate linear regression (MLR) modeling process applied to the catalyst/substrate data set identified terms relating to the steric bulk of the phosphoric acid catalyst, as expected, while the terms relating to the quinoline/pyridine and RAE were a more nuanced combination of steric and electronic factors (Scheme 3A).

The model was found to be very effective in predicting combinations of quinoline/pyridine and RAE not used in the model training, but we sought to push the boundaries by applying it to more diverse heteroarene types, previously untested in this reaction. Specifically, the model predicted that diazines, such as simple pyrimidine, should give high ee values. This prediction seemed surprising because previously for pyridines some degree of steric differentiation on the heteroarene, such as a 3-substituent, was found to be necessary to obtain high ee outcomes. However, when tested, simple pyrimidine delivered a remarkable 88% ee (predicted 89%). The model explains this positive outcome in terms of a greater NBO_*NHet*_ term due to the presence of the extra heteroatom compensating for the lower B1 term arising from the minimal sterics of the substrate. Indeed, this application to diazines proved to be very general, and a subsequent scope exploration revealed that a range of variously substituted pyrimidines gave some of the highest ee values seen for the reaction so far when an additional substitutent was included, superior to the related pyridines (Scheme 3C). We anticipate that these substrates will be of particular value in medicinal chemistry applications, and this application to pyrimidines has subsequently been utilized in the context of atroposelective Minisci reactions (see later).^20^ It is important to note that the model also effectively predicted moderately or poorly enantioselective substrates, which include RAEs whose increased length impacts ee as well as a quinazoline heterocycle (Scheme 3D).

**Scheme 4:**
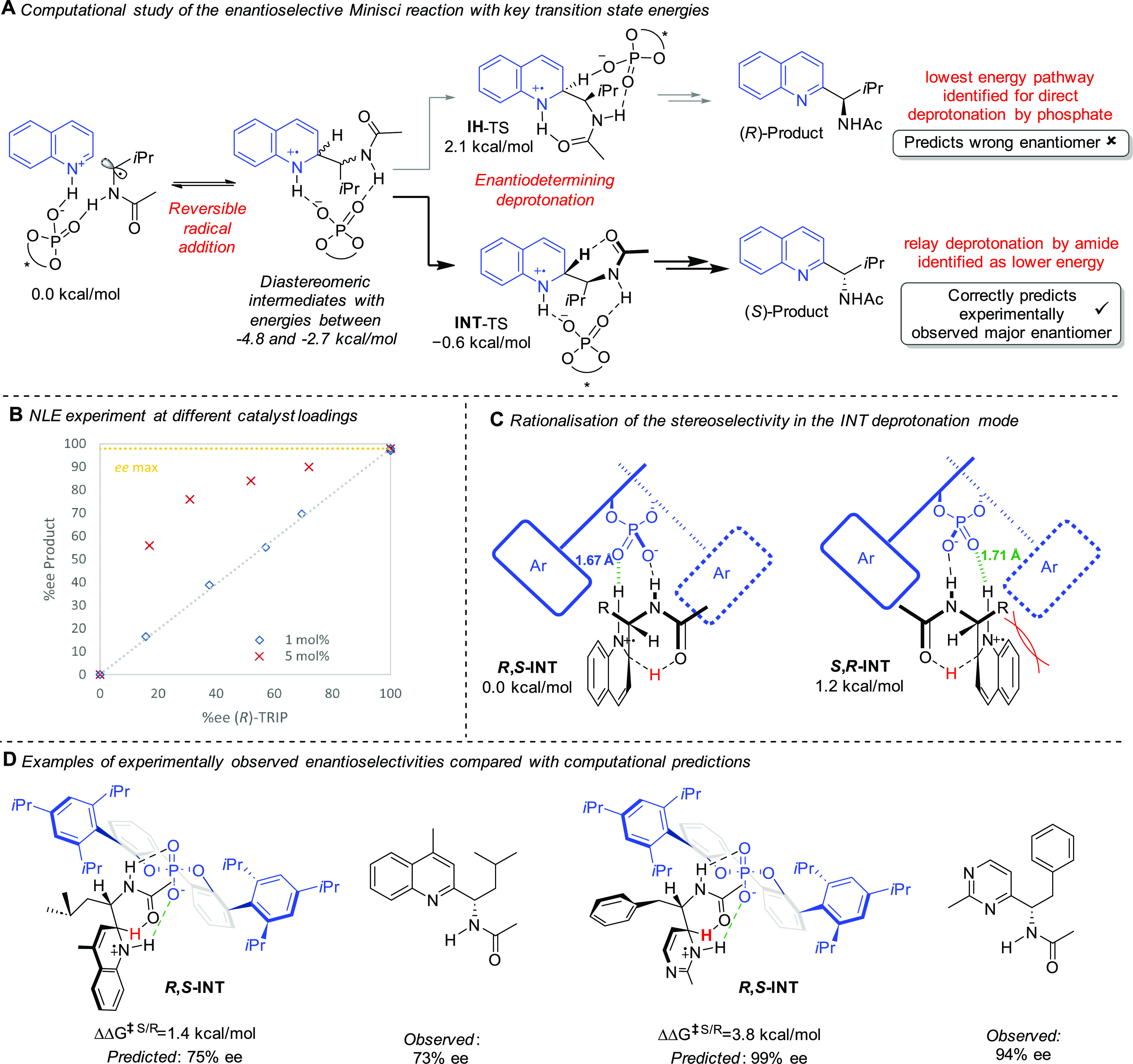
Experimental and Computational Studies to Probe the Origin of Selectivity in the Enantioselective Minisci Reaction with *N*-Acyl, α-Amino Radicals

In parallel to the above work, we had been collaborating with colleagues at the University of Cambridge, Prof. Jonathan Goodman and Dr. Kristaps Ermanis. Prof. Goodman’s group is an established leader in using DFT calculations to explore the origins of selectivity in CPA-catalyzed reactions,^21^ and they applied their expertise to interrogate the enantioselective Minisci reaction.^2^ A key question at the outset related to the positive non-linear effect (NLE) that we had previously observed and the potential implication that two molecules of a chiral catalyst may be involved in the selectivity-determining step. Due to the impact that this would have on the direction of the DFT calculations, we carried out further NLE studies and discovered that when only 1 mol % of CPA catalyst was used (as opposed to the 5% used in the previous study under the optimized experimental conditions) the non-linear effect disappeared (Scheme 4B). We carried out further experiments with a different RAE at both 5 and 1 mol % CPA loadings and observed the same; at the lower loading, there was no NLE, but at the higher one, it was present. Investigations pointed to the precipitation of a heterochiral aggregate at higher loadings, leaving the solution enhanced in one catalyst enantiomer, the visual indications of which were obscured by the limited solubility of the RAE in the dioxane solvent. This fuller investigation and identification of the reservoir effect as being responsible for the NLE allowed the computational investigations to focus on transition states involving a single molecule of TRIP.^22^

As previously described, KIE experiments suggested that the radical addition step was likely to be reversible and implied that deprotonation of the intermediate radical cation would likely constitute the product- and enantiodetermining step in a Curtin–Hammett-type mechanistic scenario. This was confirmed by calculations and a variety of plausible modes whereby the associated phosphate enacts the deprotonation of the intermediate radical cation were investigated by DFT. A mode referred to as **IH**, featuring a stabilizing internal hydrogen bond between the amide carbonyl and the quinolinium NH, was initially found to be lowest in energy (Scheme 4A, upper pathway). However, the predicted outcome was not in accordance with experiment, incorrectly predicting the *R* enantiomer as the major. Further exhaustive exploration led to the discovery of a rather unconventional deprotonation mode, **INT**, whereby the phosphate itself does not directly perform the deprotonation (Scheme 4A, lower pathway). Instead, the carbonyl of the *N*-acetyl group performs the deprotonation in an intramolecular manner, assisted by the chiral phosphate, which is associated through hydrogen bonding. This **INT** mode gave the lowest barrier so far for deprotonation and resulted in a prediction of the *S* enantiomer as the major, with a magnitude consistent with the 94% ee obtained experimentally for that substrate. An analysis of the competing transition states leading to *R* and *S* products revealed the hydrogen bond between the quinolinium and the chiral phosphate being more energetically favorable in the TS leading to the latter, with the former compromised to some degree by steric constraints, meaning that the hydrogen bond is longer and the quinolinium NH is bent further out of the quinolinium plane (Scheme 4C). The outstanding regioselectivity for the C2 position could be explained by the fact that a TS leading to C4-alkylation would be unable to invoke a hydrogen bond between the phosphate and the quinolinium NH while simultaneously assisting with the deprotonation. The model was successfully benchmarked on several other substrate combinations, including an RAE that had given a lower ee and a pyrimidine example (Scheme 4D). The insight, provided by this significant computational effort by our collaborators accounts for why the *N*-carbamoyl α-amino radicals had been so inferior to the *N*-acetyl analogues in our original optimization; they presumably are unable to enact the preferred **INT** deprotonation pathway which delivers such high selectivity.

**Figure 3:**
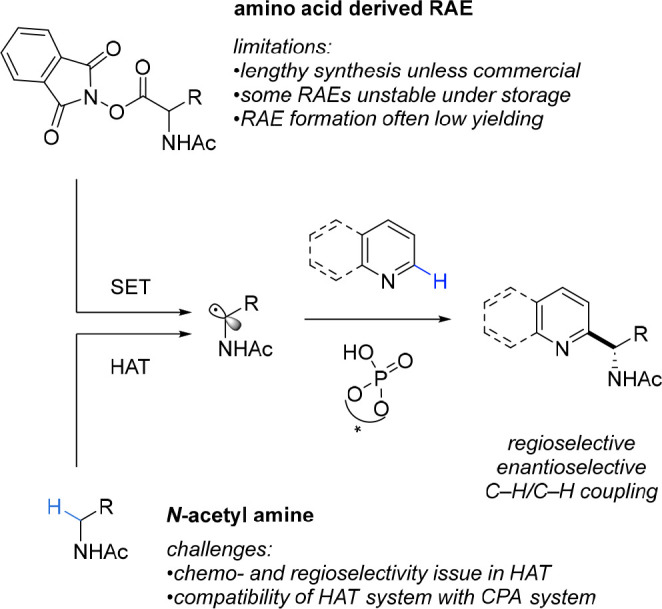
Comparison of radical generation methods *via* single electron transfer or hydrogen atom transfer.

Our originally developed protocol for the enantioselective Minisci reaction had utilized RAEs, building on the earlier non-enantioselective protocol of Shang and Fu. However, the synthesis of the RAEs necessitated starting from an amino acid, and if a desired alkyl group was not part of a readily available amino acid then a number of steps would be necessary to synthesize this. To compound this, the amino acid-derived RAEs in our hands were found to have often limited stability and shelf life, with low yields sometimes obtained for their synthesis. We supposed that, in principle, the same key *N*-acetyl, α-amino radical intermediate should be obtainable through simple hydrogen atom transfer (HAT) from the α-position of an *N*-acetylamine (Figure 3). Achieving this goal, which would constitute the formal coupling of two C–H bonds, would require a system for HAT that would not interfere with asymmetric catalysis being controlled by the CPA.

**Scheme 5:**
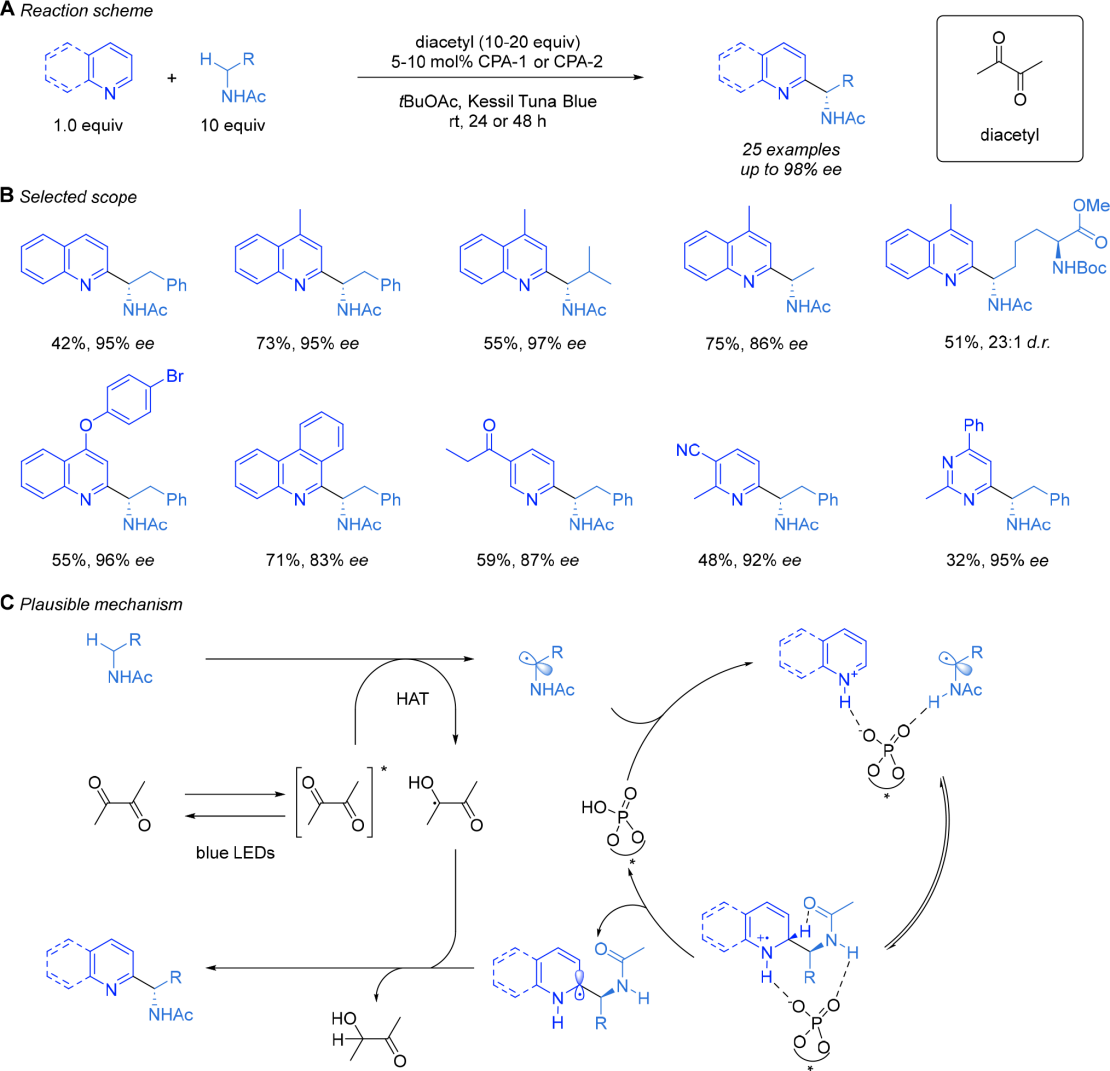
Hydrogen-Atom-Transfer-Driven Enantioselective Minisci Reaction of Amides, Selected Scope, and Proposed Mechanistic Pathway

After some experimentation, we discovered that the small molecule diacetyl worked excellently in this role. Diacetyl had been identified by Li and co-workers in 2019 as a versatile reagent capable of being directly photoexcited by blue light and, once excited, was able to perform HAT from ethers for use in Minisci reactions.^23^ Crucially, diacetyl also serves as the requisite stoichiometric oxidant for the process. Despite there being relatively few examples of HAT from the α-position of NH amides in the literature, we found that diacetyl was highly effective. Importantly, there were no compatibility issues with the CPA, and similar enantioselectivies could be obtained when compared with the RAEs in our original report (Scheme 5A).^3^ A drawback was the necessity to use 10 equiv of amide to obtain good yields, one shared in HAT-driven Minisci reactions where ether radical precursors are typically used in solvent quantities. However, we anticipate that in many cases the simplicity of the starting material when compared with having to synthesize the amino acid-derived RAE will more than compensate for this. A further advantage of this protocol is that no photocatalyst is required since diacetyl is directly photoexcited (Scheme 5C). The site selectivity for HAT adjacent to the amide was extremely high and was particularly notable given that several substrates possessed benzylic positions and tertiary alkyl C–H bonds, which could be liable to HAT. A range of quinolines and pyridines and a pyrimidine were shown to be compatible (Scheme 5B).

**Scheme 6:**
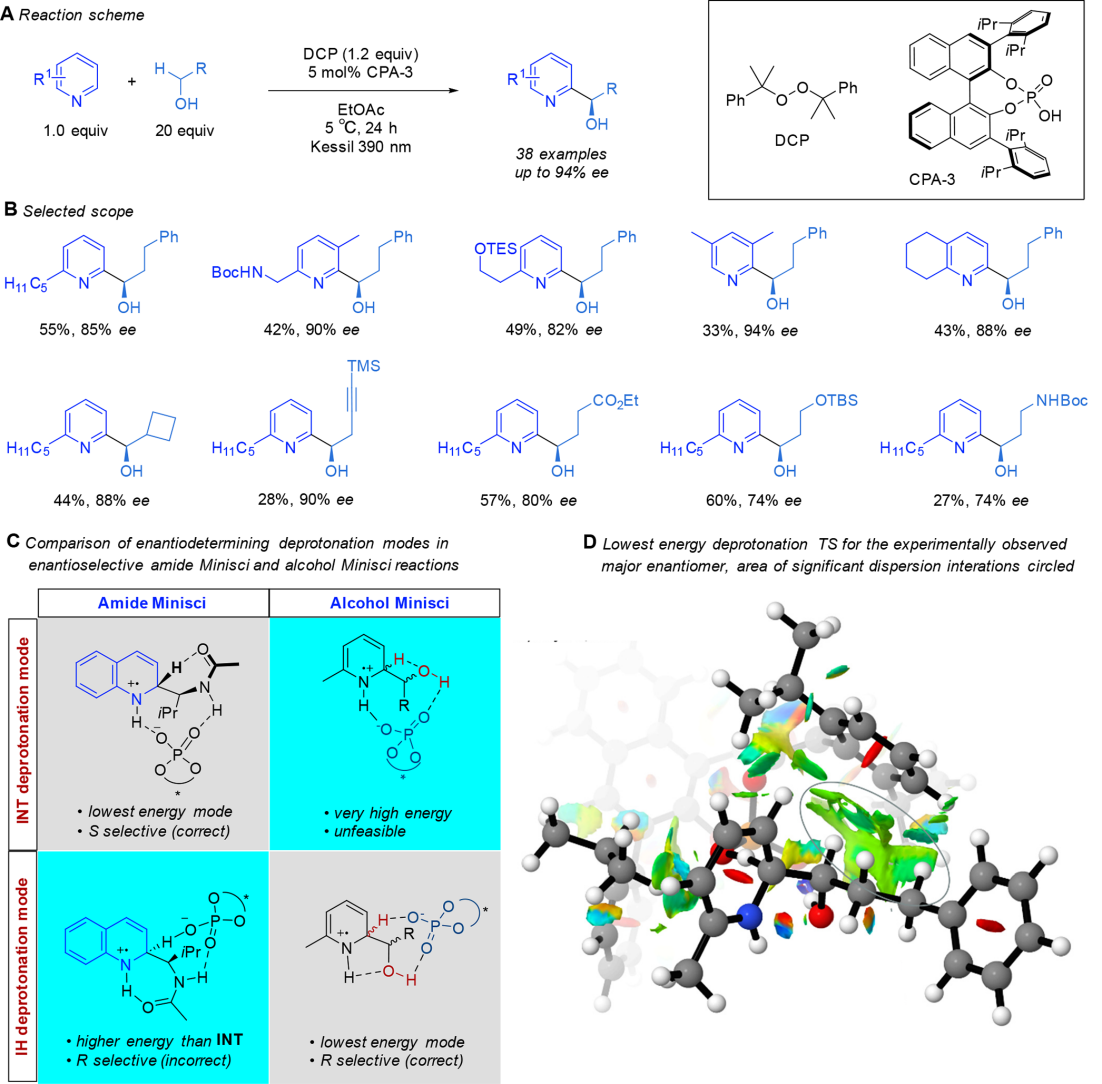
Hydrogen-Atom-Transfer-Driven Enantioselective Minisci Reaction of Alcohols, with Scope Examples and Computational Insights Panel D is reproduced with permission from ref (4). Copyright 2022 The Authors. Published by Wiley under a Creative Commons Attribution 4.0 International (CC BY 4.0) License.

In parallel to the work described above, we turned our attention to the challenge of using α-hydroxy radicals in the enantioselective Minisci reaction to generate enantioenriched secondary alcohols. It was these substrates that we had originally begun investigating before switching to the *N*-acetyl, α-amino radicals (Figure 2C, *vide supra*). While α-oxy radicals have been used extensively in Minisci reactions by way of HAT from ethers, there are fewer reports involving HAT from alcohols to produce α-hydroxy radicals. There exist a number of well-precedented side reactions that α-hydroxy radicals can undergo during Minisci reactions, including further oxidation to give an aldehyde that can itself undergo HAT to form an acyl radical able to participate in Minisci addition but to give a different product. Furthermore, upon successful addition to the heteroarene and deprotonation, the resulting neutral radical can undergo spin-center-shift elimination of water. This process has been taken advantage of in other scenarios but would be deleterious if seeking a chiral secondary alcohol product.^24^ In a push to overcome the poor yields while still obtaining enantioselectivity, we carried out an extensive optimization campaign evaluating a wide range of oxidants, HAT reagents, and photocatalysts.^4^ Use of diacetyl, as in the prior amide work, gave encouraging enantioselectivity but a low yield (23% yield, 76% ee). After much experimentation, we ultimately discovered that irradiation using a 390 nm Kessil lamp was sufficient to promote the heterolytic cleavage of dicumylperoxide (DCP), enabling it to act as a HAT reagent as well as an oxidant, with the advantage of not requiring added photocatalyst (Scheme 6A). With careful control of the reaction temperature at 5 °C and a slight modification of the CPA catalyst to use DIP (the furthest pair of isopropyl groups is removed, referred to as CPA-3 in the schemes), good levels of both yield and enantioselectivity could be obtained. We explored the scope of the reaction on a range of pyridines, and good to excellent enantioselectivities could be obtained in many cases (Scheme 6B). In some instances, yields were modest due to incomplete starting material conversion, but we found that increasing the quantity of peroxide to attempt to push conversion led to degradation and side products. Interestingly, this protocol was very specific to pyridines and failed to give useful outcomes for quinolines, instead resulting in complex mixtures. Also interesting was the observation that pyridines bearing electron-withdrawing substituents failed to react, in stark contrast with the amide Minisci where this was a requisite, the reasons for which remain unclear. A range of primary alcohols were also found to be compatible, including those with functionality such as protected amines, protected alcohols, and an alkyne. Intriguingly, the absolute configuration of the new stereocenter was found to be *R*, opposite to that obtained in the amide Minisci reaction when the same enantiomer of CPA was used. A competition experiment revealed a primary KIE of 4.4, again suggesting that deprotonation is the selectivity-determining step, just as in the amide Minisci. We were conscious that the **INT** deprotonation pathway identified as crucial by the DFT studies on the amide Minisci reaction (*vide supra*) would not be feasible with an alcohol functional group in place of an amide (Scheme 6C, upper row). Interestingly, the second-lowest-energy deprotonation mode **IH**, identified in prior work on the amide Minisci, had incorrectly predicted the *R* enantiomer as being the major. We presumed that this **IH** pathway would still be feasible for the alcohol Minisci as the hydroxy group could potentially engage in hydrogen bonding with the chiral phosphate while the phosphate itself directly enacts the deprotonation (Scheme 6C, lower row). A full DFT analysis of a range of feasible deprotonation modes by Dr. Kristaps Ermanis (by this time at the University of Nottingham) confirmed that **IH** was the lowest deprotonation mode for the alcohols, and indeed, it predicted the *R* enantiomer to be the major, consistent with experimental observations in this reaction. A detailed analysis of the transition states leading to the major and minor enantiomers suggested that extensive dispersion interactions were occurring between the substrate and the catalyst 3,3′-substituents at the TS, and a distortion–interaction analysis suggested that the difference between the major and minor transition states could be attributed to strain in the radical cation substrate, with the favored diastereomer intermediate a better fit in the catalyst pocket (Scheme 6D). Excellent agreement with experimental results was obtained through this analysis.

## Advances from Research Groups Other Than Our Own

**Scheme 7:**
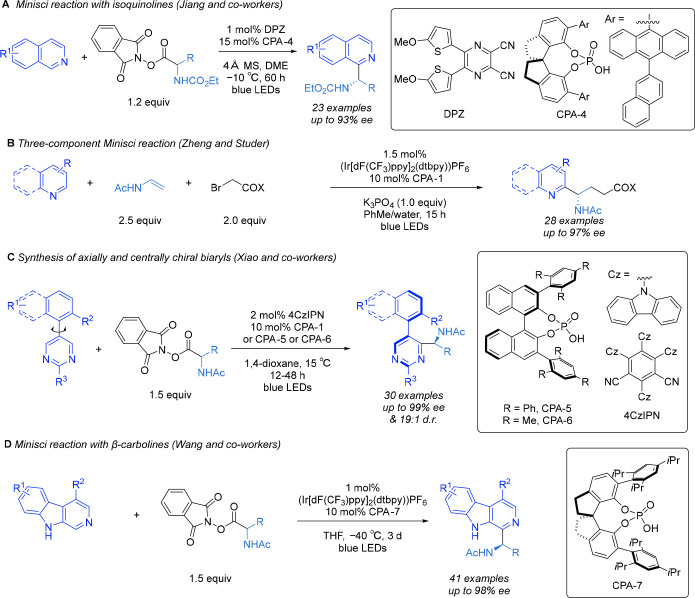
Advances in the Enantioselective Minisci Reaction from Groups Other Than Our Own

Shortly after our initial report, Jiang and co-workers published a protocol that proved effective on isoquinolines (Scheme 7A). This was an important advance as isoquinolines had given poor enantioselectivies under our original conditions.^25^ An organic photocatalyst (DPZ) was used, and SPINOL-derived phosphoric acids were crucial to obtaining the highest ee. They used a carbamate protecting group on the nitrogen of the radical with very low conversions obtained for the *N*-acetyl analogues under these conditions. Using phenylalanine-derived amino acid-derived RAEs, the scope was very good, tolerating extensive substitution on the phenylalanine arene, although removal of this aryl group proved to be detrimental to ee.

In 2019, Zheng and Studer disclosed a three-component version of the enantioselective Minisci reaction whereby the *N*-acyl, α-amino radical is constructed by the reaction of an α-bromo ester with an enamide (Scheme 7B).^26^ This is accomplished by the photocatalytic reduction of the α-bromo ester to give an electrophilic radical polarity-matched to react with the electron-rich enamide. The resulting adduct comprises an *N*-acyl, α-amino radical, as shown by us to be effective in the asymmetric Minsici reaction. This is an elegant example of increasing the complexity of the reaction mechanism without negatively affecting the asymmetric catalysis aspect.

In 2022, Xiao and co-workers applied the enantioselective Minisci protocol in an ingenious manner to enable the formation of axially chiral products through a desymmetrization process (Scheme 7C).^20^ The substrate incorporates a pyrimidine linked to a hindered, *ortho*-substituted naphthalene such that upon Minisci addition of the *N*-acyl, α-amino radical rotation of the C–C bond between the naphthalene and the pyrimidine is impossible. This process defines two stereogenic elements: one resulting from axial chirality and the other from the α-amino stereocenter. Excellent diastereo- and enantioselectivities were obtained for a range of variously substituted RAEs. Variation away from naphthalene was demonstrated, as was variation to a 3-substituted quinoline.

In a clever application of the protocol, Wang and co-workers in 2023 applied it to β-carbolines (Scheme 7D).^27^ This motif is prevalent in many alkaloids and has structural similarities to isoquinolines and pyridines. They found that SPINOL-derived phosphoric acids at low temperatures gave excellent enantioselectivity for a range of RAEs and substituted β-carbolines and showcased the reaction in the total synthesis of (+)-eudistomidin B and (+)-eudistomidin I.

**Scheme 8:**
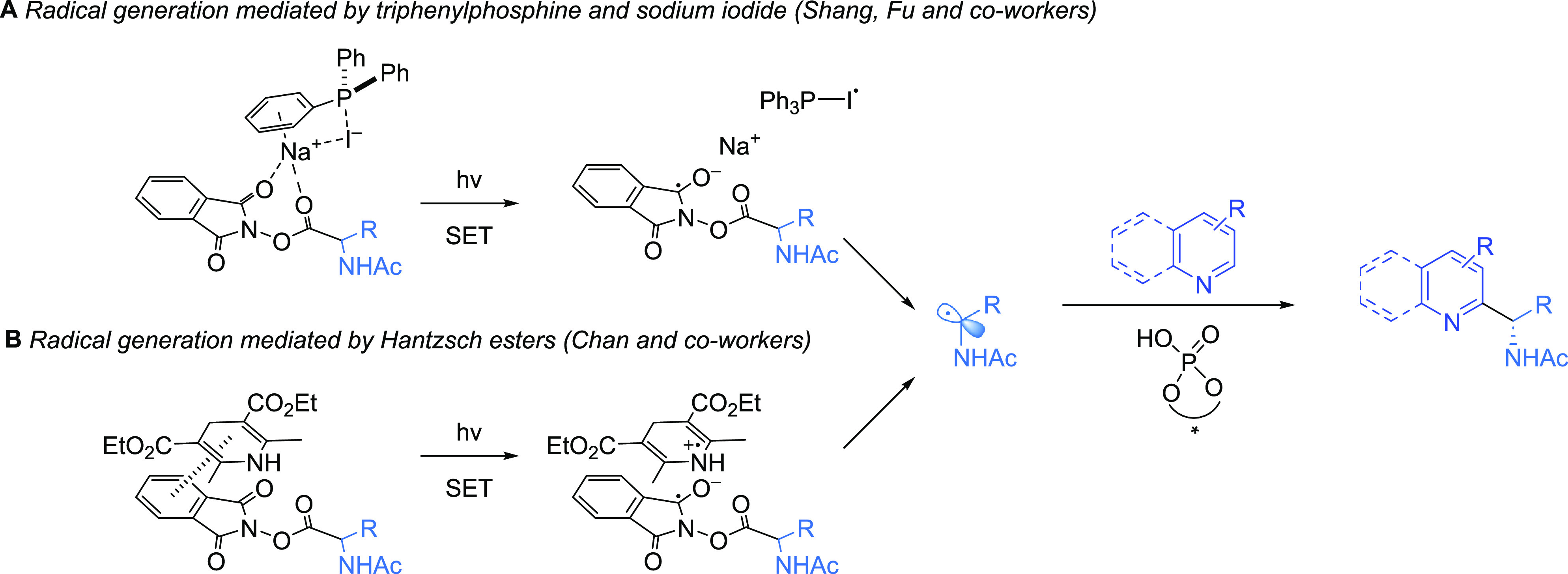
Alternative Photocatalytic Systems for RAEs in the Enantioselective Minisci Reaction

In addition to these additional developments in either radical generation or the expansion of compatible heterocyclic substrates, there have also been several reports where alternative photocatalytic systems are used in conjunction with the originally reported enantioselective Minsci reaction protocol.^1^ An impressive example comes from Shang, Fu, and co-workers, where they found that they could replace the iridium photocatalyst used in the original system with a combination of triphenylphosphine and iodine under blue LED irradiation.^28^ It is proposed that sodium iodide and triphenylphosphine associate and form a charge-transfer complex with the RAE that, upon irradiation with blue LEDs, promotes the required single electron reduction (Scheme 8A). They demonstrated this in several alkylations involving RAEs, one being the enantioselective Minisci reaction, obtaining a selection of products from our original report with similarly high levels of enantioselectivity. In 2022, Chan and co-workers developed an alternative metal-free system for the photochemical generation of alkyl radicals by way of a proposed charge-transfer complex between the RAE and Hantzsch ester (Scheme 8B).^29^ Upon irradiation with blue LEDs, electron transfer occurs to form the alkyl radical upon RAE fragmentation. This was compatible with the use of TRIP and other CPAs in the enantioselective Minisci reaction.

## Summary and Outlook

In this Account, we describe our discovery that catalytic amounts of a chiral phosphoric acid can exert control over both regioselectivity and enantioselectivity in Minisci reactions involving quinolines and pyridines, when prochiral *N*-acyl α-amino radicals are used as nucleophiles. We also describe how, since that disclosure, we have developed and investigated this reaction, often through collaboration, to better understand its mode of operation and to expand its scope and practicality. The fact that Minisci reactions inherently operate best on basic heteroarene substrates creates the valuable opportunity to use the basic heteroatom of that substrate as an interaction point with a chiral catalyst. In this way, organization during the ensuing steps of the mechanism can occur and so influence both regioselectivity and enantioselectivity. The protocol has already been inventively applied by other research groups to different substrates classes and using modified radical generation methods, which have also been mentioned in this Account. The majority of our work, as well as that of others who have also contributed to this area, has focused on α-amino radicals, but our most recent study demonstrates that this is not a necessity and that α-hydroxy radicals are also viable. We believe this is of particularly importance as it alludes to the potential wider generality of this strategy. We have very recently drawn on our experience with the Minisci reaction to enable enantioselective Giese additions of α-amino radicals by incorporating a removable pyridyl group into the substrate to enable crucial interactions with a CPA catalyst.^30^

Considering future possibilities, it is intriguing to consider whether a carefully designed CPA, perhaps with an extended structure, may enable C4-selective enantioselective Minisci reactions, something that we have so far been unable to achieve with conventional CPAs. Chiral phosphoric acids remain the most widely explored chiral Brønsted acids, but there are other types under rapid development that are more acidic and contain more confined active sites. It is possible that these may provide solutions to some outstanding challenges such as being able to activate less-basic heteroarenes or allow the addition of radicals without explicit hydrogen bond donors, with a suitably constrained cavity.^31^
